# Thrombolysis in stroke patients with elevated inflammatory markers

**DOI:** 10.1007/s00415-022-11173-0

**Published:** 2022-05-27

**Authors:** Valerian L. Altersberger, Lukas S. Enz, Gerli Sibolt, Christian Hametner, Stefania Nannoni, Mirjam R. Heldner, Jeffrey Stolp, Dejana R. Jovanovic, Andrea Zini, Alessandro Pezzini, Susanne Wegener, Carlo W. Cereda, George Ntaios, Silja Räty, Christoph Gumbinger, Miriam Heyse, Alexandros A. Polymeris, Annaelle Zietz, Anna Schaufelbuehl, Davide Strambo, Giovanna Padlina, Nedelina Slavova, Marjaana Tiainen, Kati Valkonen, Twan J. van Velzen, Guido Bigliardi, Predrag Stanarcevic, Mauro Magoni, Andreas Luft, Yannick Bejot, Laura Vandelli, Visnja Padjen, Paul J. Nederkoorn, Marcel Arnold, Patrik Michel, Peter A. Ringleb, Sami Curtze, Stefan T. Engelter, Henrik Gensicke, for the Thrombolysis in Stroke Patients (TRISP) collaborators

**Affiliations:** 1 Stroke Center and Department of Neurology, University Hospital Basel and University of Basel, Petersgraben 4, CH–4031 Basel, Switzerland; 2 Neurology, University of Helsinki and Helsinki University Hospital, Helsinki, Finland; 3 Department of Neurology, University Hospital Heidelberg, Heidelberg, Germany; 4 Department of Neurology, Service of Clinical Neurosciences, Lausanne University Hospital and University of Lausanne, Lausanne, Switzerland; 5 Department of Neurology, Inselspital, Bern University Hospital and University of Bern, Bern, Switzerland; 6 Department of Neurology, Amsterdam UMC, Location AMC, University of Amsterdam, Amsterdam, The Netherlands; 7 Neurology Clinic, Clinical Centre of Serbia, Faculty of Medicine, University of Belgrade, Belgrade, Serbia; 8 Department of Neurology and Stroke Center, IRCCS Istituto Delle Scienze Neurologiche Di Bologna, Maggiore Hospital, Bologna, Italy; 9 Department of Clinical and Experimental Sciences, Neurology Clinic, University of Brescia, Neurology Unit, ASST Spedali Civili, Brescia, Italy; 10 Department of Neurology, University Hospital Zurich and University of Zurich, Zurich, Switzerland; 11 Stroke Center and Department of Neurology, Neurocenter of Southern Switzerland, Lugano, Switzerland; 12 Department of Internal Medicine, School of Medicine, Larissa University Hospital, University of Thessaly, Larissa, Greece; 13 Department of Diagnostic and Interventional Neuroradiology and Department of Interventional, Pediatric and Diagnostic Radiology, Inselspital, Bern University Hospital and University of Bern, Bern, Switzerland; 14 Stroke Unit, Department of Neuroscience, Ospedale Civile S. Agostino-Estense, Modena University Hospital, Modena, Italy; 15 Vascular Neurology – Stroke Unit, Spedali Civili Hospital, ASST Spedali Civili, Brescia, Italy; 16 Department of Neurology, University Hospital Dijon, Dijon, France; 17 Neurology and Neurorehabilitation, Center for Medicine of Aging and Rehabilitation, University of Basel and University, Felix Platter Hospital, Basel, Switzerland

**Keywords:** Stroke, Thrombolysis, White blood cell count, CRP, Inflammation

## Abstract

**Objective:**

To investigate the prognostic value of white blood cell count (WBC) on functional outcome, mortality and bleeding risk in stroke patients treated with intravenous thrombolysis (IVT).

**Methods:**

In this prospective multicenter study from the TRISP registry, we assessed the association between WBC on admission and 3-month poor outcome (modified Rankin Scale 3–6), mortality and occurrence of symptomatic intracranial hemorrhage (sICH; ECASS-II-criteria) in IVT-treated stroke patients. WBC was used as continuous and categorical variable distinguishing leukocytosis (WBC > 10 × 10^9^/l) and leukopenia (WBC < 4 × 10^9^/l). We calculated unadjusted/ adjusted odds ratios with 95% confidence intervals (OR [95% CI]) with logistic regression models. In a subgroup, we analyzed the association of combined leukocytosis and elevated C-reactive protein (CRP > 10 mg/l) on outcomes.

**Results:**

Of 10,813 IVT-treated patients, 2527 had leukocytosis, 112 leukopenia and 8174 normal WBC. Increasing WBC (by 1 × 10^9^/l) predicted poor outcome (OR_adjusted_ 1.04[1.02–1.06]) but not mortality and sICH. Leukocytosis was independently associated with poor outcome (OR_adjusted_ 1.48[1.29–1.69]) and mortality (OR_adjusted_ 1.60[1.35–1.89]) but not with sICH (OR_adjusted_ 1.17[0.94–1.45]). Leukopenia did not predict any outcome. In a subgroup, combined leukocytosis and elevated CRP had the strongest association with poor outcome (OR_adjusted_ 2.26[1.76–2.91]) and mortality (OR_adjusted_ 2.43[1.86–3.16]) when compared to combined normal WBC and CRP.

**Conclusion:**

In IVT-treated patients, leukocytosis independently predicted poor functional outcome and death. Bleeding complications after IVT were not independently associated with leukocytosis.

**Supplementary Information:**

The online version contains supplementary material available at 10.1007/s00415-022-11173-0.

## Introduction

Systemic inflammation response is typical in patients with acute ischemic stroke (AIS) and can be caused by comorbidities (e.g., infections, malignancies or rheumatologic disorders) as well as local inflammatory processes of stroke-induced brain injury [1–3]. Inflammation enhances microvascular dysfunction and edema expansion, which ultimately can worsen the clinical outcome [4]. In clinical routine, white blood cell count (WBC) and C-reactive protein (CRP) are frequently used laboratory parameters to evaluate systemic inflammatory response [5].

In previous studies, leukocytosis (= elevated WBC) and elevated CRP were associated with more severe strokes and larger infarct volume in the general stroke population [6–9]. However, the effect of WBC and CRP on outcomes in acute stroke patients treated with IVT is contradictory. In one study, leukocytosis on admission was independently associated with poor functional outcome and occurrence of symptomatic intracranial hemorrhage (sICH) [10]. In contrast, another study did not find any association between baseline WBC and functional outcome, mortality or sICH [11]. Elevated CRP on admission was associated with poor functional outcome in one study [10] but not in another [6]. To the best of our knowledge, the combined effect of leukocytosis and elevated CRP on outcomes in IVT-treated stroke patients has not been investigated. In addition, the association of leukopenia with outcomes in this patient cohort is unknown.

With these considerations in mind, we conducted this multicenter cohort study to investigate, first, the association of WBC with outcomes in acute ischemic stroke patients treated with IVT. Second, we evaluated the prognostic importance of leukocytosis if combined with elevated CRP.

## Methods

For this cohort study, we used prospectively collected data from the international, multicenter ThRombolysis in Ischemic Stroke Patients (TRISP) collaboration, which has been described previously [12]. Thirteen TRISP centers participated in this study (Table Supplementary-1). A complete list of all TRISP centers is presented in the appendix (Appendix Table 5). Data collection was done locally in each stroke center using a standardized form with predefined variables [13]. Data of the local registries were pooled and analyzed in an anonymized way at the stroke center Basel. Variables of interest for the present study were age, sex, National Institutes of Health Stroke Scale (NIHSS) score [14], blood pressure prior to IVT treatment, onset-to-treatment time, creatinine and glucose levels, WBC and CRP in blood samples on admission, vascular risk factors according to predefined criteria [15] and prior treatment with antithrombotic agents (antiplatelet agents or anticoagulants). Outcome parameters were mortality and the modified Rankin Scale (mRS) score at 3 months assessed by either outpatient visits or telephone calls with patients and/or relatives. Poor functional outcome was defined as a mRS score of 3–6. As safety outcome, we defined the occurrence of sICH using the ECASS-II-criteria [16]. Intracranial hemorrhage was monitored by follow-up CT or MRI as described in prior research [17].

Included data were collected between June 1995 and December 2018. We excluded patients with missing data on (i) WBC on admission or (ii) 3-month outcome.

### Statistical analyses

Statistical analyses were performed with SPSS Statistics version 25 (IBM) and with R (*R Core Team (2021). R: A language and environment for statistical computing. R Foundation for Statistical Computing, Vienna, Austria. URL *https://www.R-project.org/) and RStudio (*RStudio Team (2022). RStudio: Integrated Development Environment for R. RStudio, PBC, Boston, MA URL *http://www.rstudio.com/).

We investigated the associations between WBC and outcomes using WBC as a (i) continuous variable and as a (ii) categorical variable distinguishing leukocytosis (WBC > 10 × 10^9^/l) and leukopenia (WBC < 4 × 10^9^/l) according to current hematological definitions [18, 19]. Normal WBC (4–10 × 10^9^/l) served as reference group.

Continuous data were summarized as median and interquartile range (IQR). We used chi^2^ test and Fisher’s exact test for categorical variables where appropriate and the Mann–Whitney *U* test for continuous variables. The association between WBC (± CRP) with each outcome was estimated by calculating odds ratios (OR) with 95% confidence intervals (95% CI), using binary logistic regression models. All baseline variables with Bonferroni adjusted *p* < 0.1 in univariable analyses were included in the multivariable analyses using stepwise regression with backward elimination based on the likelihood ratio test. To avoid overfitting, the maximum number of potential confounders in the final model was restricted to one-tenth of the number of outcome events, while maintaining age and NIHSS on admission as mandatory covariates in each model. Collinearity was checked in the final models by calculating the maximum generalized variance inflation factor using the car package in R, assuming a value below 5 indicating no relevant collinearity [20].

### Subgroup analyses

**Fig. 1:**
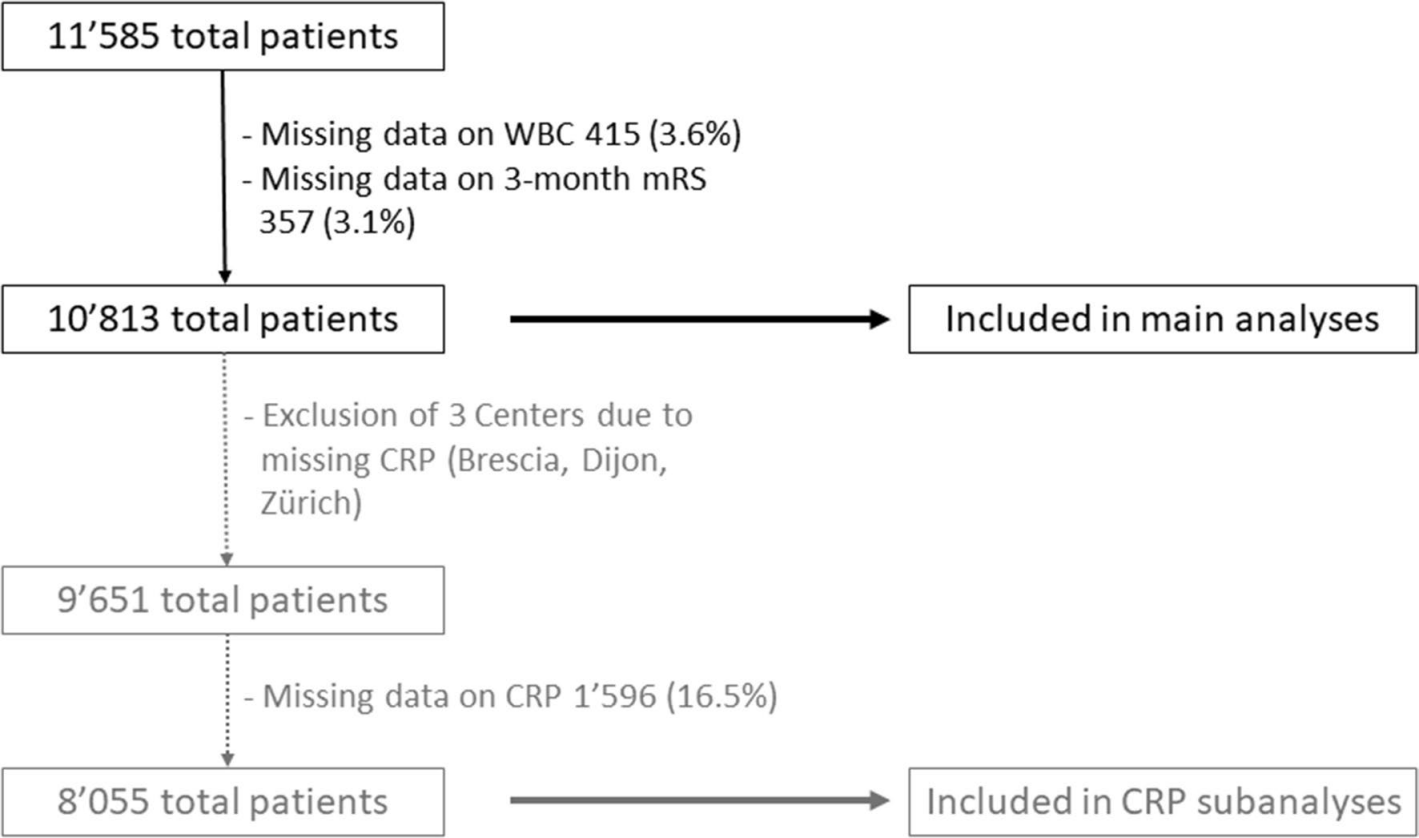
Study flowchart. Flowchart presenting the selection of eligible patients. *WBC *white blood cell count. mRS = modified Rankin Scale. *CRP*  C-reactive protein

In ten of the 13 participating centers, data on CRP on admission were available. We excluded patients from centers with missing data on CRP from the subgroup analysis (Fig. 1). In this subgroup, we assessed the association of combined leukocytosis and elevated CRP (normal range CRP < 10 mg/l [21]) on sICH, poor outcome and mortality. Patients with normal WBC and CRP served as reference group. In addition, we investigated the association between CRP and outcomes using CRP as a continuous variable.

Post hoc, we examined the association between leukocytosis and poor outcome and mortality using multivariable logistic regression models in patients admitted during three different time periods: 1995–2008, 2009–2015 and 2015–2018.

### Standard protocol approvals, registrations, and patient consents

The study was approved by the ethics committee in Basel, Switzerland and written informed consent was waived. The requirement for additional local ethical approval differed between participating centers and was obtained if required.

## Data availability statement

Anonymized data will be shared by request from any qualified investigator.

## Results

Data were eligible for analysis in 10,813 (93.3%) of the 11,585 IVT-treated patients. Reasons for exclusion were missing data on WBC (*n* = 415; 3.6%) or 3-month mRS (*n* = 357; 3.1%) (Fig. 1).

Among eligible patients, 8174 (75.6%) had normal WBC, 2527 (23.4%) leukocytosis and 112 (1.0%) leukopenia.

### Leukocytosis versus normal WBC

**Fig. 2:**
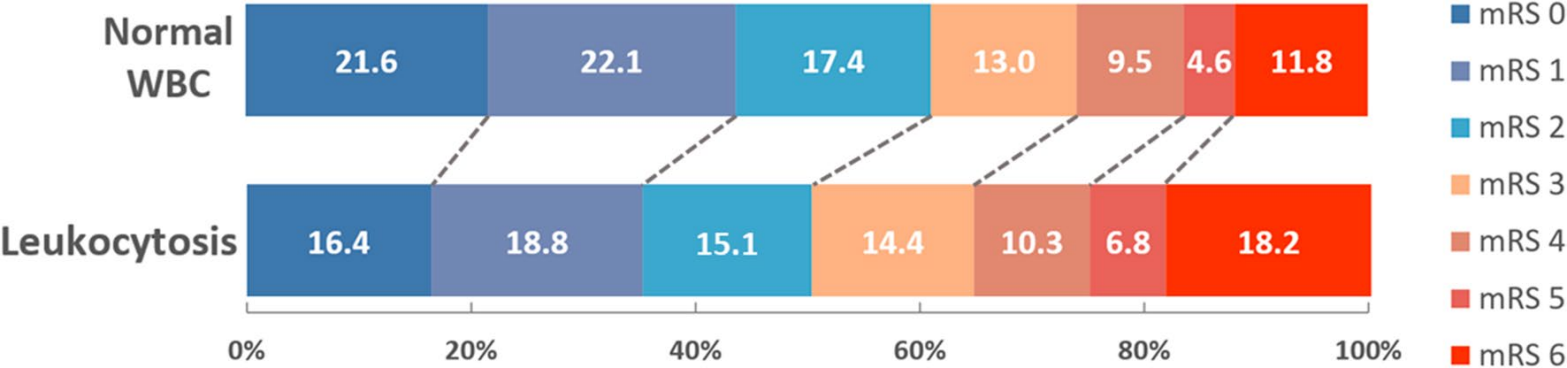
mRS at 3 months in patients with normal WBC and with leukocytosis. Modified Rankin Scale (mRS) in % at 3 months in patients with normal WBC and leukocytosis. *WBC*  white blood cell count

Baseline characteristics are presented in Table 1. Patients with leukocytosis were younger, more often active smokers, had more severe strokes on admission, longer-onset-to-treatment times and more often diabetes mellitus, prior ischemic stroke and pre-stroke disability compared to patients with normal WBC. Furthermore, patients with leukocytosis had more often poor functional outcome (49.7% vs 38.9%) and died more often (18.2% vs 11.8%) within the first 3 months and had a higher rate of sICH (5.1% vs 4.1%) (Table 1; Fig. 2).

**Table 1:** Clinical characteristics and frequency of outcome events (median (IQR) or valid percentage) of IVT-treated stroke patients divided into groups depending on their white blood cell count (WBC) at stroke onset

	Normal WBC	Leukocytosis^a^	Normal WBC vs Leukocytosis	Leukopenia^b^	Normal WBC vs Leukopenia
	*n* = *8174*	*n* = *2527*	*P* value	*n* = *112*	*P* value
WBC on admission, median (IQR)	7.3 (6.2–8.4)	11.6 (10.6–13.3)	< 0.001	3.6 (3.2–3.8)	< 0.001
Age, years, median (IQR)	72 (62–80)	70 (59–79)	< 0.001	67 (60–79)	0.058
Men, *n* (%)	4665/8174 (57.1)	1403/2527 (55.5)	0.175	61/112 (54.5)	0.631
Stroke severity, NIHSS^c^, median (IQR)	8 (5–14)	10 (6–16)	< 0.001	8.5 (5–15)	0.497
Independent prior to stroke (pre-mRS^d^ 0–2), *n* (%)	6734/7168 (93.9)	2000/2165 (92.4)	0.011	103/104 (99)	0.021
Systolic blood pressure, mmHg, median (IQR)	156 (140–173)	155 (140–173)	0.026	151 (138–168)	0.080
Onset-to-IVT time min, median (IQR)	140 (100–190)	166 (120–215)	< 0.001	140 (100–186)	0.775
Glucose on admission, mmol/l, median (IQR)	6.4 (5.7–7.6)	7.1 (6.1–8.8)	< 0.001	6.2 (5.5–7.3)	0.116
CRP^e^ on admission, mg/l, median (IQR)	3.7 (2.0–8.0)	7 (3.0–13.9)	< 0.001	3 (1.0–8.0)	0.079
Crea^f^ on admission, umol/l, median (IQR)	80 (67–96)	80 (67–99)	0.176	73 (61–88)	< 0.001
Platelets on admission, median (IQR)	215 (180–257)	250 (207–305)	< 0.001	182 (140–209)	< 0.001
Prior antithrombotics, *n* (%)	3484/7863 (44.3)	1037/2398 (43.2)	0.360	39/109 (35.8)	0.081
Atrial fibrillation, *n* (%)	2092/8111 (25.8)	631/2507 (25.2)	0.547	24/111 (21.6)	0.382
Hypertension, *n* (%)	5443/8159 (66.7)	1736/2523 (68.8)	0.053	59/112 (52.7)	0.002
Current (or stopped < 2y) Smoking, *n* (%)	1435/7036 (20.4)	645/2248 (28.7)	< 0.001	13/91 (14.3)	0.189
Hypercholesterolemia, *n* (%)	3568/8153 (43.8)	1136/2519 (45.1)	0.242	38/112 (33.9)	0.044
Diabetes mellitus, *n* (%)	1391/8152 (17.1)	572/2521 (22.7)	< 0.001	15/112 (13.4)	0.375
Coronary artery disease, *n* (%)	1557/8143 (19.1)	504/2516 (20.0)	0.312	17/112 (15.2)	0.333
Prior ischemic stroke, *n* (%)	1087/8174 (13.3)	377/2527 (15.0)	0.039	18/112 (16.1)	0.401
Any ICH^g^, *n* (%)	1078/7288 (14.8)	375/2238 (16.8)	0.024	11/102 (10.8)	0.324
Poor outcome, *n* (%)	3178/8174 (38.9)	1257/2527 (49.7)	< 0.001	44/112 (39.3)	0.923
Mortality, *n* (%)	962/8174 (11.8)	460/2527 (18.2)	< 0.001	16/112 (14.3)	0.378
Symptomatic ICH (ECASS-2 criteria), *n* (%)	338/8031 (4.2)	130/2472 (5.3)	0.028	5/112 (4.5)	0.812

^a^Leukocytosis: WBC > 10 × 109/l, ^b^leukopenia: WBC < 4 × 109/l, ^c^*NIHSS* national institutes of health stroke scale, ^d^*mRS* modified rankin scale, ^e^*CRP* C-reactive protein, ^f^*Crea* Creatinine, ^g^*ICH* intracerebral hemorrhage

In unadjusted logistic regression analyses, leukocytosis increased the odds of poor functional outcome (OR_unadjusted_ 1.56, 95% CI 1.42–1.70), mortality (OR_unadjusted_ 1.66, 95% CI 1.47–1.88) and sICH (OR_unadjusted_ 1.26, 95% CI 1.02–1.55) (Table 2). After adjusting for potential confounders, the association of poor outcome (OR_adjusted_ 1.48, 95% CI 1.29–1.69) and mortality (OR_adjusted_ 1.60, 95% CI 1.35–1.89) with leukocytosis remained (Table 3). Using the same adjusted model, the association between leukocytosis and poor outcome and mortality remained stable over the long time period of data collection: (i) 1995–2008: poor outcome OR_adjusted_ 1.44, 95% CI 1.10–1.89; mortality OR_adjusted_ 1.57, 95% CI 1.12–2.19; (ii) 2009–2015: poor outcome OR_adjusted_ 1.43, 95% CI 1.21–1.69; mortality 1.51, 95% CI 1.22–1.86; (iii) 2015–2018: poor outcome OR_adjusted_ 2.07, 95% CI 1.33–3.21; mortality OR_adjusted_ 2.17, 95% CI 1.35–3.48 (Table Supplementary-3). Leukocytosis increased the odds for sICH in the unadjusted (OR_unadjusted_ 1.26, 95% CI 1.02–1.55) but not in the adjusted analyses (OR_unadjusted_ 1.17, 95% CI 0.94–1.45) (Tables 2 and 3). The maximum variance inflation factor for this model was 1.19, suggesting no relevant collinearity disturbing the model.

**Table 2:** Putative predicting variables. Odds ratio (95% confidence interval), adj. p-value^1^

Putative predicting variables	sICH	Poor outcome	Mortality
Age (each year)	1.02 (1.02–1.03), adj.*p* < 0.001	1.05 (1.05–1.05), adj.*p* < 0.001	1.07 (1.06–1.07), adj.*p* < 0.001
Female sex	0.81 (0.67–0.97), adj.*p* = 1	0.68 (0.63–0.73), adj.*p* < 0.001	0.73 (0.65–0.82), adj.*p* < 0.001
NIHSS (each point)	1.07 (1.06–1.08), adj.*p* < 0.001	1.18 (1.18–1.19), adj.*p* < 0.001	1.15 (1.14–1.16), adj.*p* < 0.001
Systolic blood pressure (each 10 mmHg)	1.00 (1.00–1.01), adj.*p* = 1	1.00 (1.00–1.00), Trend^2^: + , adj.*p* = 0.399	1.00 (1.00–1.00), Trend^2^: + , adj.*p* = 1
Onset-to-IVT time min (each minute)	1.00 (1.00–1.00), Trend^2^: + , adj.*p* = 1	1.00 (1.00–1.00), Trend^2^: + , adj.*p* = 0.004	1.00 (1.00–1.00), Trend^2^: + , adj.p = 0.197
Creatinine (by 1 umol/l)	1.00 (1.00–1.00), Trend^2^: + , adj.*p* = 1	1.00 (1.00–1.00), Trend^2^: + , adj.*p* < 0.001	1.01 (1.00–1.01), adj.*p* < 0.001
Glucose (each mmol/l)	1.07 (1.04–1.10), adj.*p* < 0.001	1.12 (1.10–1.14), adj.*p* < 0.001	1.11 (1.09–1.13), adj.*p* < 0.001
Independent prior to stroke (pre-mRS 0–2)	0.68 (0.48–0.99), adj.*p* = 1	0.07 (0.06–0.10), adj.*p* < 0.001	0.22 (0.18–0.26), adj.*p* < 0.001
Prior antithrombotics	1.58 (1.31–1.91), adj.*p* < 0.001	1.58 (1.46–1.71), adj.*p* < 0.001	1.97 (1.75–2.21), adj.*p* < 0.001
Atrial fibrillation	1.54 (1.26–1.87), adj.*p* < 0.001	2.01 (1.84–2.19), adj.*p* < 0.001	2.12 (1.88–2.38), adj.*p* < 0.001
Hypertension	1.35 (1.10–1.67), adj.*p* = 0.272	1.55 (1.43–1.69), adj.*p* < 0.001	1.53 (1.35–1.74), adj.*p* < 0.001
Current (or stopped < 2y) Smoking	0.58 (0.43–0.77), adj.*p* = 0.014	0.68 (0.61–0.75), adj.*p* < 0.001	0.49 (0.41–0.58), adj.*p* < 0.001
Hypercholesterolemia	1.06 (0.88–1.28), adj.*p* = 1	0.89 (0.83–0.96), adj.*p* = 0.208	0.91 (0.81–1.02), adj.*p* = 1
Diabetes mellitus	1.36 (1.08–1.68), adj.*p* = 0.373	1.74 (1.58–1.92), adj.*p* < 0.001	1.66 (1.46–1.89), adj.*p* < 0.001
Coronary artery disease	1.39 (1.12–1.72), adj.*p* = 0.158	1.41 (1.28–1.56), adj.*p* < 0.001	1.90 (1.67–2.15), adj.*p* < 0.001
Prior ischemic stroke	1.24 (0.96–1.58), adj.*p* = 1	1.49 (1.34–1.67), adj.*p* < 0.001	1.58 (1.37–1.83), adj.*p* < 0.001
Leukocytosis (vs normal WBC)	1.26 (1.02–1.55), adj.*p* = 1	1.56 (1.42–1.70), adj.*p* < 0.001	1.66 (1.47–1.88), adj.*p* < 0.001
Leukopenia (vs normal WBC)	1.00 (0.35–2.23), adj.*p* = 1	0.91 (0.62–1.33), adj.*p* = 1	1.09 (0.62–1.80), adj.*p* = 1
Increasing WBC (by 1)	1.00 (0.99–1.00), adj.*p* = 1	1.07 (1.05–1.08),adj.*p* < 0.001	1.00 (1.00–1.01), adj.*p* = 1

^1^Whole table adjusted by Bonferroni’s method

^2^If numerically unclear, trend indicates the direction of association: “ + ” indicates a positive relation (i.e., mean larger than 1), “– “ a negative relation (i.e., mean smaller than 1)

**Table 3:** Multivariable analysis of outcomes. Odds ratio (95% confidence interval), *p*-value

Putative predicting variables	Outcome measures		
	sICH	Poor outcome	Mortality
Leukocytosis vs normal WBC	1.17 (0.94–1.45)^1^ *p* = 0.164	1.48 (1.29–1.69)^3^ *p* < 0.001	1.60 (1.35–1.89)^4^ *p* < 0.001
Leukopenia vs normal WBC	1.02 (0.35–2.30)^2^ *p* = 0.969	1.05 (0.66–1.65)^2^ *p* = 0.827	1.32 (0.35–2.30)^2^ *p* = 0.379
Increasing WBC (by 1 × 10^9^/l)	1.00 (0.98–1.00)^1^ *p* = 0.721	1.04 (1.02–1.06)^3^ *p* < 0.001	1.00 (1.00–1.01)^4^ *p* = 0.096

^1^Adjusted for: age, NIHSS on admission, glucose on admission, prior antithrombotics

^2^Adjusted for: age, NIHSS on admission

^3^Adjusted for: age, gender, NIHSS on admission, glucose on admission, independence prior to stroke, prior ischemic stroke

^4^Adjusted for: age, NIHSS on admission, creatinine on admission, glucose on admission, independence prior to stroke, prior antithrombotics, coronary artery disease

### Leukopenia versus normal WBC

Compared to normal WBC, leukopenia (*n* = 112; 1.04%) did not significantly change the odds for any outcome (poor functional outcome OR_adjusted_ 1.05, 95% CI 0.66–1.65; mortality OR_adjusted_ 1.32, 95% CI 0.69–2.38; sICH OR_adjusted_ 1.02, 95% CI 0.35–2.30) (Table 3).

### WBC as a continuous variable

Increasing WBC was associated with poor outcome (increase by 1 × 10^9^/l, OR_unadjusted_ 1.07, 95% CI 1.05–1.08) but not with mortality (OR_unadjusted_ 1.00, 95% CI 1.00–1.01) and sICH (OR_unadjusted_ 1.00, 95% CI 0.99–1.00) (Table 3). After adjusting for potential confounders the association of poor outcome (OR_adjusted_ 1.04, 95% CI 1.02–1.06) with WBC remained. The maximum variance inflation factor for this model was 1.06, suggesting no relevant collinearity disturbing the model.

### Subgroup of patients with available information on CRP and WBC

Out of 9651 patients, data on CRP were available in 8055 patients. (Fig. 1). Of those, 622 (7.7%) patients had elevated CRP combined with leukocytosis, whereas WBC and CRP were normal in 5′177 (64.3%) patients. Baseline characteristics of these two groups are presented in Table Supplementary-2. Poor outcome (64.0% vs 36.5%) and mortality (28.5% vs 10.3%) were more frequent in patients with combined leukocytosis and elevated CRP.

Combined leukocytosis and elevated CRP was independently associated with poor functional outcome (OR_unadjusted_ 3.07, 95% CI 2.53–3.73 and OR_adjusted_ 2.26, 95% CI 1.76–2.91) and mortality (OR_unadjusted_ 3.86, 95% CI 3.09–4.81 and OR_adjusted_ 2.43, 95% CI 1.86–3.16) but not with sICH compared to normal WBC and CRP (Table 4).

**Table 4:** CRP subgroup analyses. Multivariable analysis of outcomes. Odds ratio (95% confidence interval), *p*-value

Putative predicting variables	Outcome measures		
	sICH	Poor outcome	Mortality
Elevated CRP and leukocytosis (vs normal WBC and normal CRP)	1.03 (0.69–1.52)^1^ *p* = 0.881	2.26 (1.76–2.91)^3^ *p* < 0.001	2.43 (1.86–3.16)^5^ *p* < 0.001
Increasing CRP (by 1 mg/l)	1.00 (0.99 -1.01)^2^ *p* = 0.786	1.01 (1.01–1.02)^4^ *p* < 0.001	1.01 (1.01–1.02)^5^ *p* < 0.001

^1^Adjusted for: age, NIHSS on admission

^2^Adjusted for: age, NIHSS on admission, glucose on admission, prior antithrombotics

^3^Adjusted for: age, NIHSS on admission, glucose on admission, independence prior to stroke, prior ischemic stroke

^4^Adjusted for: age, gender, NIHSS on admission, glucose on admission, independence prior to stroke, prior ischemic stroke

^5^Adjusted for: age, NIHSS on admission, creatinine on admission, glucose on admission, independence prior to stroke, coronary artery disease

In the subgroup analysis, increasing CRP (by 1 mg/l) was associated with poor outcome (OR_adjusted_ 1.01, 95% CI 1.01–1.02) and mortality (OR_adjusted_ 1.01, 95% CI 1.01–1.02) but not with sICH (OR_adjusted_ 1.00, 95% CI 0.99 -1.01) (Table 4).

## Discussion

Key results for the association between white blood cell counts (WBC) and outcomes in acute ischemic stroke patients treated with IVT were: (i) Leukocytosis on admission independently predicted poor functional outcome and mortality. This association was even more pronounced in the subgroup of patients with combined leukocytosis and elevated CRP. (ii) Neither leukocytosis nor the combination of leukocytosis and elevated CRP was significantly associated with the occurrence of sICH. (iii) Leukopenia was not associated with any outcome.

Previous studies revealed an association between leukocytosis as well as elevated CRP on admission and more severe strokes and larger infarct volumes [6–9]. In line, patients with leukocytosis in the present study had higher baseline NIHSS than patients with normal WBC (median NIHSS 10 vs 8). This difference was even more pronounced in the subgroup of patients with combined leukocytosis and elevated CRP (median NIHSS 12 vs 8) suggesting that the extent of brain injury might — at least partly — be reflected by inflammatory parameters. Another explanation for the higher stroke severity in patients with elevated inflammatory parameters might be that those patients had significantly longer time intervals between stroke onset and administration than patients with normal WBC (median 166 min vs 140 min). Despite the fact, that our study was not designed to investigate potential causes of treatment delay, these associations stress the importance of investigating outcomes in this patient cohort. Yet, the effect of leukocytosis on outcomes and hemorrhagic complications in IVT-treated stroke patients in previous studies was controversial. One study, including 985 IVT-treated stroke patients, found an association between baseline leukocytosis and poor outcome [10], whereas in another study, including 846 IVT-treated stroke patients, baseline neutrophil count but not leukocytosis independently predicted poor outcome and mortality [11]. A third study found baseline WBC < 8.1 × 10^9^/l to be associated with favorable outcome in IVT-treated stroke patients (*n* = 657) and suggested further investigation of WBC in larger prospective datasets [22]. Limitation of these studies is the relatively small number of patients with leukocytosis. In the present study, we found an independent association between leukocytosis and poor functional outcome and mortality, which remained stable over the long period of data collection (1995–2018). Interestingly, association with poor functional outcome and mortality was even stronger in patients with leukocytosis and elevated CRP. Additionally, the probability for poor functional outcome and mortality increased with rising WBC and CRP. However, every increase in WBC by 1 × 10^9^/l increased the odds of poor functional outcome by 4% which is likely of minor clinical relevance. While we cannot evaluate the cause of the association between elevated inflammatory parameters and worse clinical outcome, it is likely that elevated inflammatory parameters contribute directly –e.g., via leucocyte clogging [23] — and indirectly — reflecting a co-existing disorder — to outcome after IVT.

Because patients with laboratory signs of systemic inflammation had higher NIHSS at onset and larger stroke volumes [6–9], it would have been likely that hemorrhagic complications after thrombolysis (sICH) occur more often in patients with laboratory signs of systemic inflammation than in those without. Accordingly, experimental research found that leukocytes contribute to vascular damage and disruption of the blood–brain barrier [24, 25]. Furthermore, one previous study revealed an independent association between baseline leukocytosis and occurrence of sICH [10] and another recent study investigating 510 ischemic stroke patients (196 treated with IVT) suggested an association of high WBC with parenchymal hemorrhage independent of infections [26]. However, in our study, after adjustment for potential confounders, neither leukocytosis nor the combination of leukocytosis and elevated CRP was independently associated with increased rate of sICH. Because increased baseline NIHSS, onset-to-treatment time and glucose levels on admission — all well-established risk factors of sICH — were more often present in patients with leukocytosis, we assume that the unadjusted association of leukocytosis with sICH is mostly due to these confounding parameters. Therefore, it seems unlikely that the association between elevated systemic inflammation parameters and poor outcome and mortality is caused by sICH.

Leukopenia — as defined by hematologic criteria — has not been investigated in IVT-treated stroke patients prior to this study. Numerous causes of leukopenia are likely to be associated with worse outcome (e.g., (viral) infections, (hematotoxic) drugs, autoimmune disorders, radiation, renal failure, malignancies, malnutrition) [27, 28]. Additionally, lymphopenia was shown to independently predict unfavorable outcome in intracerebral hemorrhage [29]. In our study, however, presence of leukopenia on admission was rare (1.0%) and did not change the odds for any outcome suggesting that leukopenia is rather a chance finding than a manifestation of an outcome modifying disorder in this patient cohort.

Strengths of our study are the large sample size (*n* = 10,813) which allowed adjusting for multiple confounding variables and thus clarifying the inconsistencies of previous studies. We also included a subgroup analysis with combined leukocytosis and elevated CRP which is a novelty and underlined the effect of systemic inflammation on clinical outcomes. Finally, we were able to assess outcomes in patients with leukopenia.

The present study has limitations apart from general limitations of register-based, retrospective studies: (i) causes of leukocytosis and elevated CRP were unclear and information on disorders that are likely associated with systemic inflammatory response (e.g., infections at admission, malignancies, rheumatologic disorders or certain medication) was not collected and it was not possible to determine whether leukocytosis was a trigger for stroke. However, as patients suffering from chronic infections or cancer are likely to have pre-existing disability, we were at least able to partly address this issue by adjusting for pre-stroke mRS. (ii) There is growing evidence on the strong linkage between ischemia and inflammation/ immunity [30, 31], and immunomodulation agents are evaluated for acute stroke therapy and stroke prevention [32, 33]. Yet, the observational design of our study does not allow conclusions on potential therapeutic measures modulating the inflammatory response. (iii) Leukocyte subclasses were not available. Thus, we were not able to examine the previously described association of neutrophils/ neutrophil-to-lymphocyte ratio with poor outcome, mortality and sICH [11] in our patient cohort. In addition, the association between leukopenia and outcomes was difficult to interpret as red blood cell and platelet counts were not available.

### Electronic supplementary material

Below is the link to the electronic supplementary material.

Supplementary file1 (DOCX 17 KB)

## Appendix

See below Table

**Table 5:** TRISP collaborators (alphabetic order by city name)

Name	Location	Role	Contribution
Paul Nederkoorn, MD, PhD	Amsterdam UMC	Global Principal Investigator Site Principal Investigator	Co-founder of TRISP Designs and conceptualizes studies Analyzes and interprets data Drafts manuscripts Revises manuscripts Collects data
Yvo Roos, MD, PhD	Amsterdam UMC	Site Investigator	Revises manuscripts Collects data
Sophie van den Berg, MD	Amsterdam UMC	Site Investigator	Revises manuscripts Collects data
Lotte Stolze	Amsterdam UMC	Site Investigator	Analyzes and interprets data Drafts manuscripts Revises manuscripts Collects data
Thomas Zonneveld, MD	Amsterdam UMC	Site Investigator	Analyzes and interprets data Drafts manuscripts Revises manuscripts Collects data
Leon Rinkel, MD	Amsterdam UMC	Site Investigator	Revises manuscripts Collects data
Stefan Engelter, MD	University Hospital, Basel	Global Principal Investigator	Co-founder of TRISP Designs and conceptualizes studies Analyzes and interprets data Drafts manuscripts Revises manuscripts Collects data
Henrik Gensicke, MD	University Hospital, Basel	Site Principal Investigator Global Network Coordinator	Leads and coordinates communication among sites Designs and conceptualizes studies Analyzes and interprets data Drafts manuscripts Revises manuscripts Collects data
Valerian Altersberger, MD	University Hospital, Basel	Site Investigator	Designs and conceptualizes studies Analyzes and interprets data Drafts manuscripts Revises manuscripts Collects data
Lukas Simon Enz, MD, PhD	University Hospital, Basel	Site Investigator	Analyzes and interprets data Revises manuscripts
Leo Bonati, MD	University Hospital, Basel	Site Investigator	Revises manuscripts Collects data
Gian Marco De Marchis, MD, MSc	University Hospital, Basel	Site Investigator	Revises manuscripts Collects data
Philippe Lyrer, MD	University Hospital, Basel	Site Investigator	Revises manuscripts Collects data
Alexandros Polymeris, MD	University Hospital, Basel	Site Investigator	Revises manuscripts Collects data
Marios Psychogios, MD	University Hospital, Basel	Site Investigator	Revises manuscripts Collects data
Alex Brehm, MD	University Hospital, Basel	Site Investigator	Revises manuscripts Collects data
Visnja Padjen, MD, PhD	Clinical Centre of Serbia, Belgrade	Site Principal Investigator	Designs and conceptualizes studies Revises manuscripts Collects data
Dejana Jovanovic, MD, PhD	Clinical Centre of Serbia, Belgrade	Site Investigator	Collects data Revises manuscripts
Ivana Berisavac, MD, PhD	Clinical Centre of Serbia, Belgrade	Site Investigator	Collects data Revises manuscripts
Marko Ercegovac, MD, PhD	Clinical Centre of Serbia, Belgrade	Site Investigator	Revises manuscripts
Predrag Stanarcevic, MD	Clinical Centre of Serbia, Belgrade	Site Investigator	Collects data Revises manuscripts
Maja Budimkic, MD, PhD	Clinical Centre of Serbia, Belgrade	Site Investigator	Collects data
Ljiljana Bumbasirevic, MD, PhD	Clinical Centre of Serbia, Belgrade	Site Investigator	Revises manuscripts
Christian Nolte, MD	University Hospital Charite, Berlin	Site Principal Investigator	Designs and conceptualizes studies Analyzes and interprets data Drafts manuscripts Revises manuscripts Collects data
Hebun Erdur, MD	University Hospital Charite, Berlin	Site Investigator	Designs and conceptualizes studies Analyzes and interprets data Drafts manuscripts Revises manuscripts Collects data
Jan Scheitz, MD	University Hospital Charite, Berlin	Site Investigator	Designs and conceptualizes studies Analyzes and interprets data Drafts manuscripts Revises manuscripts Collects data
Marcel Arnold, MD	University Hospital, Bern	Site Principal Investigator	Designs and conceptualizes studies Analyzes and interprets data Drafts manuscripts Revises manuscripts Collects data
Mirjam Heldner, MD, MSc	University Hospital, Bern	Site Principal Investigator	Designs and conceptualizes studies Analyzes and interprets data Drafts manuscripts Revises manuscripts Collects data
Urs Fischer, MD, MSc	University Hospital, Bern	Site Investigator	Interprets data Drafts manuscripts Revises manuscripts Collects data
Simon Jung, MD	University Hospital, Bern	Site Investigator	Analyzes and interprets data Drafts manuscripts Revises manuscripts Collects data
Roland Wiest, MD	University Hospital, Bern	Site Investigator	Interprets data Drafts manuscripts Revises manuscripts Collects data
Andrea Zini, MD	IRCCS Istituto delle Scienze Neurologiche, Bologna	Site Principal Investigator	Analyzes and interprets data Drafts manuscripts Revises manuscripts Collects data
Mauro Gentile, MD	IRCCS Istituto delle Scienze Neurologiche, Bologna	Site Investigator	Revises manuscripts Collects data
Andreas Kastrup	Bremen	Site Principal Investigator	Interprets data Collects data Revises manuscripts
Panagiotis Papanagiotou	Bremen	Site Investigator	Interprets data Collects data Revises manuscripts
Alessandro Pezzini, MD	ASST Spedali Civili, Brescia	Site Principal Investigator	Collects data Revises manuscripts
Mauro Magoni, MD	ASST Spedali Civili, Brescia	Site Investigator	Collects data Revises manuscripts
Alessandro Padovani, MD, PhD	ASST Spedali Civili, Brescia	Site Investigator	Collects data Revises manuscripts
Yannick Béjot, MD, PhD	University Hospital, Dijon	Site Principal Investigator	Designs and conceptualizes studies Analyzes and interprets data Drafts manuscripts Revises manuscripts Collects data
Maurice Giroud, MD, PhD	University Hospital, Dijon	Site Investigator	Collects data
Guy-Victor Osseby, MD	University Hospital, Dijon	Site Investigator	Collects data
Marie Hervieu-Bègue, MD	University Hospital, Dijon	Site Investigator	Collects data
Christelle Blanc-Labarre, MD	University Hospital, Dijon	Site Investigator	Collects data
Benoit Delpont, MD	University Hospital, Dijon	Site Investigator	Collects data
Annika Nordanstig	University Hospital, Gothenburg	Site Investigator	Analyzes and interprets data Drafts manuscripts Revises manuscripts Collects data
Jan-Erik Karlsson, MD, PhD	University Hospital, Gothenburg	Site Investigator	Revises manuscripts Collects data
Camilla Karlsson, MD	University Hospital, Gothenburg	Site Investigator	Analyzes and interprets data Revises manuscripts Collects data
Alexandros Rentzos, MD, PhD	University Hospital, Gothenburg	Site Investigator	Revises manuscripts Collects data
Katarina Jood, MD, PhD	University Hospital, Gothenburg	Site Investigator	Revises manuscripts Collects data
Turgut Tatlisumak, MD, PhD	University Hospital, Gothenburg	Site Principal Investigator	Designs and conceptualizes studies Analyzes and interprets data Drafts manuscripts Revises manuscripts Collects data
Jan Liman, MD	University medical center, Göttingen	Site Principal Investigator	Revises manuscripts Collects data
Peter Ringleb, MD	University Hospital, Heidelberg	Site Principal Investigator	Designs and conceptualizes studies Analyzes and interprets data Drafts manuscripts Revises manuscripts Collects data
Christian Hametner, MD, MSc	University Hospital, Heidelberg	Site Investigator	Designs and conceptualizes studies Analyzes and interprets data Drafts manuscripts Revises manuscripts Collects data
Timolaos Rizos, MD	University Hospital, Heidelberg Alfried Krupp Hosptial, Essen	Site Investigator	Designs and conceptualizes studies Revises manuscripts Collects data
Mona Laible, MD	University Hospital, Heidelberg	Site Investigator	Designs and conceptualizes studies Drafts manuscripts Revises manuscripts Collects data
Anne Berberich, MD	University Hospital, Heidelberg	Site Investigator	Revises manuscripts Collects data
Christoph Gumbinger, MD	University Hospital, Heidelberg	Site Investigator	Revises manuscripts Collects data
Sami Curtze, PhD, MD, MSc	University Hospital, Helsinki	Site Principal Investigator	Designs and conceptualizes studies Analyzes and interprets data Drafts manuscripts Revises manuscripts Collects data
Nicolas Martinez-Majander, MD	University Hospital, Helsinki	Site Investigator	Designs and conceptualizes studies Analyzes and interprets data Drafts manuscripts Revises manuscripts Collects data
Jukka Putaala, PhD, MD, MSc	University Hospital, Helsinki	Site Investigator	Designs and conceptualizes studies Analyzes and interprets data Drafts manuscripts Revises manuscripts Collects data
Silja Räty, MD, MSc	University Hospital, Helsinki	Site Investigator	Designs and conceptualizes studies Analyzes and interprets data Drafts manuscripts Revises manuscripts Collects data
Gerli Sibolt, MD, MSc	University Hospital, Helsinki	Site Investigator	Designs and conceptualizes studies Analyzes and interprets data Drafts manuscripts Revises manuscripts Collects data
Daniel Strbian, PhD, MD, MSc	University Hospital, Helsinki	Site Investigator	Designs and conceptualizes studies Analyzes and interprets data Drafts manuscripts Revises manuscripts Collects data
Marjaana Tiainen, PhD, MD, MSc	University Hospital, Helsinki	Site Investigator	Designs and conceptualizes studies Analyzes and interprets data Drafts manuscripts Revises manuscripts Collects data
Kati Valkonen, MD	University Hospital, Helsinki	Site Investigator	Revises manuscripts Collects data
Ronen Leker, MD	Hadassah-Hebrew University Medical Center, Jerusalem	Site Principal Investigator	Designs and conceptualizes studies Analyzes and interprets data Drafts manuscripts Revises manuscripts Collects data
Asaf Honig, MD	Hadassah-Hebrew University Medical Center, Jerusalem	Site Investigator	Analyzes and interprets data Revises manuscripts Collects data
Andrei Filioglo, MD	Hadassah-Hebrew University Medical Center, Jerusalem	Site Investigator	Analyzes and interprets data Revises manuscripts Collects data
Jose E. Cohen, MD	Hadassah-Hebrew University Medical Center, Jerusalem	Site Investigator	Analyzes and interprets data Revises manuscripts Collects data
Georges Ntaios, MD, PhD	University of Thessaly, Larissa	Site Principal Investigator	Analyzes and interprets data Revises manuscripts Collects data
Patrik Michel, MD	University Hospital, Lausanne	Site Principal Investigator	Analyzes and interprets data Collects data Revises manuscripts
Stefania Nannoni, MD	University Hospital, Lausanne	Site Investigator	Collects data Revises manuscripts
Davide Strambo, MD	University Hospital, Lausanne	Site Investigator	Collects data Revises manuscripts Analyzes and interprets data
Gaia Sirimarco, MD, PhD	University Hospital, Lausanne	Site Investigator	Collects data Revises manuscripts
Marie Bodenant, MD	University Hospital, Lille	Site Investigator	Collects data Revises manuscripts
Charlotte Cordonnier, MD, PhD	University Hospital, Lille	Site Principal Investigator	Collects data Revises manuscripts
Nelly Dequatre-Ponchelle, MD	University Hospital, Lille	Site Investigator	Collects data Revises manuscripts
Hilde Hénon, MD, PhD	University Hospital, Lille	Site Investigator	Collects data Revises manuscripts
Yaohua Chen, MD	University Hospital, Lille	Site Investigator	Collects data Revises manuscripts
Solène Moulin, MD, MSc	University Hospital, Lille	Site Investigator	Collects data Revises manuscripts
Carlo Cereda, MD	Neurocenter of Southern Switzerland, Lugano	Site Principal Investigator	Designs and conceptualizes studies Analyzes and interprets data Drafts manuscripts Revises manuscripts Collects data
Giovanni Bianco, MD	Neurocenter of Southern Switzerland, Lugano	Site Investigator	Analyzes and interprets data Collects data Revises manuscripts
Guido Bigliardi, MD	University Hospital, Modena	Site Principal Investigator	Collects data Revises manuscripts Analyzes and interprets data
Laura Vandelli, MD	University Hospital, Modena	Site Investigator	Collects data Revises manuscripts
Maria Dell’Acqua, MD	University Hospital, Modena	Site Investigator	Collects data Revises manuscripts
Francesca Rosafio, MD	University Hospital, Modena	Site Investigator	Collects data Revises manuscripts
Livio Picchetto, MD	University Hospital, Modena	Site Investigator	Collects data Revises manuscripts
Stefania Maffei, MD, PhD	University Hospital, Modena	Site Investigator	Collects data Revises manuscripts
Stefano Meletti, MD	University Hospital, Modena	Site Investigator	Collects data Revises manuscripts
Valentina Fioravanti, MD	University Hospital, Modena	Site Investigator	Collects data Revises manuscripts
Giuseppe Borzì, MD	University Hospital, Modena	Site Investigator	Collects data Revises manuscripts
Riccardo Ricceri, MD	University Hospital, Modena	Site Investigator	Collects data Revises manuscripts
Lars Kellert, MD	Ludwig Maximilians University, Munich	Site Principal Investigator	Designs and conceptualizes studies Analyzes and interprets data Drafts manuscripts Revises manuscripts Collects data
Katharina Feil, MD	Ludwig Maximilians University, Munich	Site Investigator	Collects data Analyzes and interprets data Drafts manuscripts
Clemens Küpper, MD	Ludwig Maximilians University, Munich	Site Investigator	Collects data Analyzes and interprets data Drafts manuscripts
Johanna Heinrich, MD	Ludwig Maximilians University, Munich	Site Investigator	Collects data Analyzes and interprets data Drafts manuscripts
Marialuisa Zedde, MD	Azienda USL-IRCCS, Reggio Emilia	Site Principal Investigator	Collects data Analyzes and interprets data Revises manuscripts
Jean-Louis Mas, MD	Centre Hospitalier Sainte-Anne, Paris	Site Investigator	Collects data Revises manuscripts
Catherine Oppenheim, MD, PhD	Centre Hospitalier Sainte-Anne, Paris	Site Investigator	Collects data Revises manuscripts
Pierre Seners, MD, MSc	Centre Hospitalier Sainte-Anne, Paris	Site Investigator	Collects data Revises manuscripts
Guillaume Turc, MD, PhD	Centre Hospitalier Sainte-Anne, Paris	Site Principal Investigator	Collects data Revises manuscripts
Bruno Gonçalves, MD	Centre Hospitalier Sainte-Anne, Paris	Site Principal Investigator	Collects data Revises manuscripts
Georg Kaegi, MD	Kantonsspital St. Gallen	Site Principal Investigato	Analyzes and interprets data Drafts manuscripts Revises manuscripts Collects data
Susanne Wegener, MD	University Hospital, Zurich	Site Principal Investigator	Designs and conceptualizes studies Analyzes and interprets data Drafts manuscripts Revises manuscripts Collects data
Laura Westphal, MD	University Hospital, Zurich	Site Investigator	Analyzes and interprets data Drafts manuscripts Collects data
Philipp Baumgartner, MD	University Hospital, Zurich	Site Investigator	Analyzes and interprets data Revises manuscripts
Andreas Luft, MD	University Hospital, Zurich	Site Investigator	Analyzes and interprets data Revises manuscripts

5 here.
