# Supplementary material for: Thrombolysis in stroke patients with elevated inflammatory markers
Source: J Neurol. 2022 May 27;269(10):5405–19. doi: 10.1007/s00415-022-11173-0 (PMC9468078; doi:10.1007/s00415-022-11173-0)
Supplement: Supplementary file 1 — Supplementary file1 (DOCX 17 KB) [file 415_2022_11173_MOESM1_ESM.docx]

**Supplemental material**

**Table e-1. Participating TRISP centers.**

| Center (city, country) | Period | IVT, n |
| --- | --- | --- |
| Amsterdam, the Netherlands | 01/2000 – 12/2014 | 635 |
| Basel, Switzerland | 06/1998 – 12/2017 | 1406 |
| Belgrade, Serbia | 02/2006 – 10/2017 | 503 |
| Bern, Switzerland | 03/2000 – 12/2017 | 949 |
| Brescia, Italy | 02/2010 – 05/2017 | 268 |
| Dijon, France | 02/2007 – 12/2012 | 410 |
| Heidelberg, Germany | 03/1998 – 03/2017 | 1954 |
| Helsinki, Finland* | 06/1995 – 12/2017 | 3598 |
| Larissa, Greece | 02/2014 – 12/2018 | 37 |
| Lausanne, Switzerland | 01/2003 – 12/2017 | 1083 |
| Lugano, Switzerland | 01/2014 – 12/2017 | 127 |
| Modena, Italy | 05/2005 – 12/2012 | 421 |
| Zürich, Switzerland | 01/2014 – 09/2016 | 194 |
| Total | 06/1995 – 12/2018 | 11585 |

* Part of this population was also used in Tiainen M et al. Int J Stroke. 2013 Dec;8(8):632-8.

**Table e-2.** CRP subanalysis. Clinical characteristics and frequency of outcome events of IVT-treated stroke patients divided into groups depending on their white blood cell count (WBC) and C-reactive protein (CRP) at stroke onset.

|  | **Leukocytosis^a^ and elevated CRP^b^** | **Normal WBC and CRP** | **Normal CRP and WBC**  **vs**  **Leukocytosis and elevated CRP** |
| --- | --- | --- | --- |
|  | *n=622* | *n=5’177* | P value |
| WBC on admission, median (IQR) | 12 (10.8-13.5) | 7.2 (6.1-8.3) | <0.001 |
| CRP on admission, mg/l, median (IQR) | 3 (2-5) | 24 (14-54) | <0.001 |
| Age, years, median (IQR) | 74 (63-82) | 72 (61-79) | <0.001 |
| Men, n (%) | 309/622 (49.7) | 2991/5177 (57.8) | <0.001 |
| Stroke severity, NIHSS^c^, median (IQR) | 12 (7-18) | 8 (5-14) | <0.001 |
| Independent prior to stroke (pre-mRS^d^ 0-2), n (%) | 447/522 (85.6) | 4427/4699 (94.2) | <0.001 |
| Systolic blood pressure, mmHg, median (IQR) | 155 (137-173) | 157 (140-174) | 0.013 |
| Onset-to-treatment, min, median (IQR) | 165 (125-213) | 138 (100-185) | <0.001 |
| Glucose on admission, mmol/l, median (IQR) | 7.3 (6.3-9.0) | 6.4 (5.6-7.5) | <0.001 |
| Crea^e^ on admission, umol/l, median (IQR) | 83 (66-107) | 80 (68-94) | 0.003 |
| Prior antithrombotics, n (%) | 305/618 (49.4) | 2264/5167 (43.8) | 0.009 |
| Atrial fibrillation, n (%) | 211/617 (34.2) | 1311/5133 (25.5) | <0.001 |
| Hypertension, n (%) | 466/621 (75.0) | 3452/5170 (66.8) | <0.001 |
| Current (or stopped < 2y) Smoking, n (%) | 140/555 (25.2) | 871/4275 (20.4) | 0.008 |
| Hypercholesterolemia, n (%) | 269/619 (43.5) | 2331/5169 (45.1) | 0.439 |
| Diabetes mellitus, n (%) | 179/619 (28.9) | 832/5166 (16.1) | <0.001 |
| Coronary artery disease, n (%) | 153/618 (24.8) | 936/5159 (18.1) | <0.001 |
| Prior ischemic stroke, n (%) | 110/621 (17.7) | 706/5163 (13.7) | 0.006 |
| Any ICH^f^ | 87/539 (16.1) | 636/4612 (13.8) | 0.137 |
| Poor outcome | 398/622 (64.0) | 1892/5177 (36.5) | <0.001 |
| Mortality | 177/622 (28.5) | 531/5177 (10.3) | <0.001 |
| Symptomatic ICH (ECASS-2 criteria) | 31/609 (5.1) | 196/5072 (3.9) | 0.144 |

**Legend**

a Leukocytosis: WBC (white blood cell count) > 10x109/l, b elevated CRP (C-reactive protein): CRP >10mg/l c NIHSS: National Institutes of Health Stroke Scale, d mRS: modified Rankin Scale, e Crea: Creatinine, f ICH: intracerebral haemorrhage

**Table e-3.** Multivariable analysis of poor outcome and mortality in patients with leucocytosis on admission during three time periods (1995-2008, 2009-2015 and 2015-2018). Odds ratio (95% confidence interval), p-value.

| **Leukocytosis vs normal WBC** | **Poor outcome** | **Mortality** |
| --- | --- | --- |
| 1995-2008; n= 2’803 | 1.44 (1.10-1.89)^1^ p=0.009 | 1.57 (1.12-2.19)^2^ p=0.008 |
| 2009-2015; n= 6’333 | 1.43 (1.21-1.69)^1^ p<0.001 | 1.51 (1.22-1.86)^2^ p<0.001 |
| 2015-2018; n= 1’621 | 2.07 (1.33-3.21)^1^ p=0.001 | 2.17 (1.35-3.48)^2^ p=0.001) |

1 Adjusted for: age, gender, NIHSS on admission, glucose on admission, independence prior to stroke, prior ischemic stroke

2 Adjusted for: age, NIHSS on admission, creatinine on admission, glucose on admission, independence prior to stroke, prior antithrombotics, coronary artery disease
